# Cataract GWAS meta-analysis and multi-omics Mendelian randomisation inform disease mechanisms and candidate target prioritisation

**DOI:** 10.1016/j.ebiom.2026.106485

**Published:** 2026-09-22

**Authors:** Xuanming Hong, Linxuan Zhang, Verena Zuber, Zihao Zhuang, Héléne T. Cronjé, Stephen Burgess, Dipender Gill

**Affiliations:** a Department of Epidemiology and Biostatistics, School of Public Health, Imperial College London, London, UK; b Department of Epidemiology and Biostatistics, School of Public Health, Peking University, Beijing, China; c MRC Biostatistics Unit, School of Clinical Medicine, University of Cambridge, Cambridge, UK; d The Second Affiliated Hospital of Fujian Medical University, Quanzhou, Fujian, China; e Sequoia Genetics, London, UK

**Keywords:** Cataract, Genome-wide association study, Meta-analysis, Target identification, Pleiotropy

## Abstract

**Background:**

Cataract is a highly prevalent cause of visual impairment and blindness worldwide. Although genome-wide association studies (GWAS) have mapped multiple cataract risk loci, further work is required towards translating these signals and integrating multi-omics evidence for preventing or delaying age-related cataract progression.

**Methods:**

A senile cataract GWAS was first conducted in All of Us, followed by ancestry-specific GWAS meta-analyses in European-ancestry (161,812 cases and 1,099,738 controls) and East Asian ancestry (66,679 cases and 521,995 controls). To prioritise candidate targets, we performed Mendelian randomisation (MR) and colocalisation using cis-expression quantitative trait loci (cis-eQTL) instruments from eQTLGen. We then conducted a plasma proteome-wide cis-pQTL MR scan with colocalisation using UKB-PPP and deCODE for European analyses, and cis-pQTL data from the China Kadoorie Biobank for East Asian analyses.

**Findings:**

We identified 78 and 4 independent loci for cataract in Europeans and East Asians, respectively, including 8 and 3 putatively unreported loci. By integrating the meta-GWAS with cis-eQTL MR, we prioritised 16 genes, including *MTMR3*, *NT5DC2*, and *EFNA1*, with eQTL-GWAS colocalisation further supporting *MTMR3* and *NT5DC2*. Proteome-wide cis-pQTL MR prioritised 32 proteins for senile cataract in Europeans, and colocalisation refined these to *EFNA1* and *RALY*. In East Asians, MR highlighted *APOE* and *ALDH2* as candidate targets. We also identified colocalised pleiotropic signals shared between cataract and related traits, including myopia, hypertension, and type 2 diabetes, highlighting candidate genes such as *TCF7L2*, *BMP4*, *CDKAL1*, *PAX4*, and *ALDH2*.

**Interpretation:**

These findings advance cross-ancestry understanding of cataract genetic architecture and prioritise genetically supported candidates.

**Funding:**

This work was supported by the Joint Funds for the Innovation of Science and Technology, Fujian Joint Innovation Fund in Science and Technology Project (Grant No. 2025Y9437).

**Research in context**

**Evidence before this study**

Cataract is a leading cause of visual impairment and blindness worldwide, and age-related cataract accounts for most cases in older adults. Although cataract surgery is effective, pharmacological strategies to prevent or delay cataract progression remain limited. Previous genome-wide association studies have identified multiple cataract susceptibility loci, including loci with functional relevance to lens biology. However, most large-scale studies have been conducted predominantly in European-ancestry populations, with comparatively fewer data from East Asian populations. In addition, previous cataract GWAS have largely focused on locus discovery, with less systematic integration of transcriptomic, proteomic, and cross-trait genetic evidence to prioritise biologically plausible candidates.

**Added value of this study**

We conducted a senile cataract GWAS in the All of Us and performed ancestry-stratified GWAS meta-analyses in European and East Asian populations. The European meta-analysis identified 78 independent loci, including 8 putatively unreported loci, while the East Asian meta-analysis identified 4 independent loci, including 3 putatively unreported loci. Cross-ancestry comparison suggested moderate sharing of cataract genetic architecture while highlighting ancestry-relevant signals. By integrating GWAS findings with cis-eQTL MR, proteome-wide cis-pQTL MR, colocalisation, and pleiotropy analyses, we prioritised multiple genetically supported candidates, including *MTMR3*, *NT5DC2*, *EFNA1*, *RALY*, *APOE*, *ALDH2*, and shared pleiotropic genes linking cataract with myopia, hypertension, and type 2 diabetes.

**Implications of all the available evidence**

These findings expand the genetic map of cataract across European and East Asian populations and provide a framework for moving from GWAS locus discovery towards biologically informed genetic prioritisation. The prioritised genes and proteins implicate pathways involving lens homoeostasis, oxidative stress, glucose regulation, and shared cardiometabolic biology. Larger harmonised GWAS, ocular tissue QTL resources, and experimental studies are needed to refine mechanisms and evaluate their potential for preventing or delaying age-related cataract progression.

## Introduction

Cataract is characterised by progressive opacification of the crystalline lens, leading to visual impairment and potentially blindness.^1^ Its occurrence increases with age, making ageing the primary risk factor. Age-related (senile) cataract accounts for the majority of cases in older adults, with approximately 18% of individuals older than 60 years affected.^2^^,^^3^ Globally, estimates for 2020 suggest that cataract accounted for about 15.2 million cases of blindness and 78.8 million cases of moderate-to-severe vision impairment, underscoring its substantial public health burden.^4^

Cataract can be effectively treated by surgical removal of the opacified lens with implantation of an artificial intraocular lens, yet financial barriers, limited surgical capacity, and unequal access to care result in many people in need of surgery being unable to receive it.^2^ Moreover, pharmacological strategies to prevent or delay age-related cataract progression remain limited.^5^ Identifying genetic variants that influence cataract susceptibility therefore has important clinical implications, as it can improve etiologic understanding and help prioritise genetically supported pathways for prevention and therapeutic development. Large-scale genome-wide association studies (GWAS) have mapped dozens of loci associated with cataract risk.^6^^,^^7^ Importantly, several loci implicate genes with direct functional evidence and translational potential. For example, *EPHA2* has been associated with age-related cortical cataract in humans, and Epha2 knockout mice develop progressive cataracts, supporting a causal role in lens opacity in vivo.^8^ These findings continue to motivate genetic and mechanistic studies aimed at prevention and therapeutic target discovery.

In addition, cataract commonly co-occurs with cardiometabolic and other ocular disorders. A recent review has systematically summarised these frequent comorbidities, suggesting shared underlying biology.^9^ This comorbidity pattern provides a rationale for leveraging large-scale GWAS to delineate shared genetic architecture and prioritise pleiotropic, potentially druggable cataract targets. One illustrative example is *AKR1B1*, which encodes aldose reductase in the polyol pathway and has long been considered a tractable target in diabetic cataract. In animal studies, the aldose reductase inhibitor ranirestat markedly suppressed cataract development in diabetic rat models, providing pharmacologic evidence that this pathway is modifiable.^10^

In this work, we performed a GWAS of senile cataract in the All of Us (AoU) research program cohort, and then conducted ancestry-stratified GWAS meta-analyses of cataract in European and East Asian participants to advance understanding of its genetic architecture. We then integrated prioritised association signals with cis-expression quantitative trait loci (cis-eQTL) instruments and applied Mendelian randomisation (MR) to evaluate gene expression-mediated effects at mapped loci, thereby prioritising putative effector genes. Next, we leveraged cis-protein quantitative trait loci (cis-pQTL) instruments to perform a blood-based proteome-wide cis-pQTL MR as a systematic discovery step to nominate additional, genetically supported candidates for cataract. Following this, informed by the shared genetic basis between cataract and related ocular, cardiometabolic, and ageing-related traits, we used complementary pleiotropy and colocalisation analyses to further refine and biologically contextualise prioritised candidates.

## Methods

### All of Us population

We first conducted a GWAS using data from the All of Us Research Program, a large-scale, longitudinal cohort study launched by the U.S. National Institutes of Health. Details of the cohort design, recruitment, consent framework, and participant characteristics have been described previously.^11^ All participants provided written informed consent for the broad research use of their health and genetic data. The present analysis was conducted within the All of Us Researcher Workbench using the Controlled Tier Curated Data Repository (CDR) version 8.

### Phenotype definition

Senile cataract cases were ascertained from electronic health record (EHR) data using the All of Us Cohort Builder, based on Systematised Nomenclature of Medicine-Clinical Terms (SNOMED) standard concept identifiers descending from the parent concept “Senile cataract” together with the corresponding mapped source codes from the International Classification of Diseases, Ninth and Tenth Revisions (ICD-9 and ICD-10). Cases were defined as participants with at least one occurrence of a qualifying diagnosis code in the EHR. This EHR-based definition captures clinically recorded senile cataract but is not equivalent to slit-lamp-graded cataract or a specific morphological subtype. Controls were defined as participants with no record of any cataract diagnosis in the EHR and with at least one EHR contact. The complete list of SNOMED concept identifiers and ICD source codes used is provided in Supplementary Table S1.

### Genomic data of All of Us

We restricted analyses to participants of genetically inferred European ancestry, as assigned by the All of Us centralised ancestry classifier.^12^ The analytic sample comprised 317,969 participants, including 40,087 senile cataract cases and 277,882 controls. Whole-genome sequencing data were obtained from the short-read whole-genome sequencing (srWGS) ACAF threshold callset in the All of Us Controlled Tier Dataset version 8. Details of the sequencing pipeline, joint variant calling, and centralised quality control are described in Bick et al.^12^ and Supplementary Methods.

### Additional GWAS data source and phenotyping of cataract

The GWAS meta-analysis was first conducted for senile (age-related) cataract in a total of 1,261,550 participants of European ancestry, including 161,812 cases and 1,099,738 controls. Summary statistics were obtained from two sources. The first source was the senile cataract GWAS meta-analysis reported by Diaz-Torres et al., which combined 4 European cohorts, including the UK Biobank (UKB), the Genetic Epidemiology Research in Adult Health and Ageing (GERA) cohort, the Mass General Brigham Biobank (MGBB), and FinnGen, comprising 121,725 cataract cases and 821,856 controls (total n = 943,581). In that study, cataract status was primarily ascertained from electronic health records using ICD-9/ICD-10 diagnosis and/or procedure codes in GERA, MGBB, and FinnGen. In UKB, cases were additionally identified based on self-reported cataract operation (field 20004, code 1435) and/or hospital records with cataract diagnoses (ICD-10 H25 or H26), and the remaining participants were treated as controls. In FinnGen, the outcome corresponded to the senile cataract endpoint in release eight. Detailed cohort-specific phenotype definitions, genotyping, quality control, and imputation procedures have been described previously by Diaz-Torres et al.^7^ The second source was the newly conducted AoU GWAS of senile cataract for the present study, including 40,087 cases and 277,882 controls of European genetic ancestry. To further validate the putatively novel lead variants identified in AoU GWAS and the European meta-GWAS, we additionally examined their associations in the Million Veteran Program (MVP) GWAS of senile cataract (PheCode 366.2) which included 197,600 cases and 206,486 controls (total n = 404,086).^13^ To improve comparability with the meta GWAS and reduce potential confounding from population stratification, the MVP summary statistics were restricted to individuals of European genetically inferred ancestry (Supplementary Table S2). Given these related but non-identical definitions, the European meta-GWAS should be interpreted as predominantly reflecting age-related cataract rather than a uniformly defined morphological subtype.

A large GWAS meta-analysis was also conducted in individuals of East Asian ancestry, including 66,679 cases and 521,995 controls. Summary statistics were derived from three major biobank resources: the Taiwan Precision Medicine Initiative (TPMI: 27,180 cases and 306,565 controls), BioBank Japan (BBJ: 38,194 cases and 140,532 controls), and the China Kadoorie Biobank (CKB: 1305 cases and 74,898 controls). Detailed cohort descriptions have been reported previously. In TPMI, cataract cases were defined using PheCode 366.2, consistent with the definition applied in MVP.^14^ In CKB, age-related cataract was defined using ICD-10 code H25.^15^ In BBJ, cataract was likewise defined using ICD-10 code H25, although cataract subtypes were not further distinguished.^16^ Accordingly, the Asian-ancestry meta-analysis was interpreted as cataract, largely reflecting age-related (senile) cataract, rather than a strictly harmonised senile-cataract endpoint across all contributing cohorts (Supplementary Table S2).

### GWAS data sources for cataract-related traits

GWAS summary statistics for cataract-related traits and comorbidities were leveraged to evaluate shared genetic architecture and pleiotropic loci with cataract. These phenotypes spanned ocular traits (myopia and uveitis), cardiometabolic traits (hypertension and type 2 diabetes), and ageing-related phenotypes represented by epigenetic clocks (Hannum, GrimAge, and PhenoAge).^9^ In European ancestry, GWAS summary statistics for myopia, uveitis, and hypertension were obtained from the MVP.^13^ For type 2 diabetes mellitus (T2DM), ancestry-specific meta-analysis summary statistics reported by Suzuki et al. were used as the primary datasets, with the European-ancestry analysis aggregating 33 cohorts and the Asian-ancestry analysis aggregating 25 cohorts.^17^ Since these primary T2DM GWAS datasets may include partial sample overlap with the cataract meta-GWAS, we performed sensitivity analyses using an MVP T2DM GWAS for European ancestry and a Korean Genome and Epidemiology Study (KoGES) diabetes GWAS for East Asian ancestry.^18^ For East Asian ancestry, hypertension GWAS summary statistics were also obtained from KoGES. Since clinically defined myopia GWAS data independent of the cataract meta-GWAS were unavailable in East Asians, refractive error GWAS summary statistics from Cheng et al. were used as a proxy for myopia. This study included 5 East Asian cohorts and did not include BBJ, CKB, or TPMI.^19^ Reported estimates were retained on the original refractive-error scale, with lower values indicating greater myopic liability. Because no alternative East Asian GWAS of uveitis was available, TPMI uveitis summary statistics were retained only for supportive analysis.^14^ Detailed characteristics of each GWAS, including case and control counts, are provided in the Supplementary Materials and Supplementary Table S3.

To capture ageing-related biology potentially relevant to cataract, GWAS summary statistics for three epigenetic clock traits were additionally incorporated. These data were available only in European ancestry and were obtained from the study by McCartney et al., which analysed 34,710 European-ancestry participants and examined genetic determinants of multiple DNA methylation-based biomarkers.^20^

In selecting GWAS datasets for cataract-related traits, we prioritised ancestry-matched summary statistics and avoided overlap with the primary cataract meta-GWAS where feasible.

### Protein and gene expression quantitative trait loci data

To investigate the effects of target genes on cataract, we leveraged large-scale protein quantitative trait loci (pQTL) and expression quantitative trait loci (eQTL) resources. For plasma protein instruments in European ancestry, two large pQTL datasets were used: the deCODE proteomics study (n = 35,559) and the UK Biobank Pharma Proteomics Project (UKB-PPP, n = 46,281).^21^^,^^22^ Protein levels were measured using the SomaScan platform in deCODE and the Olink platform in UKB-PPP, as described in the original publications. After harmonising protein identifiers across platforms and removing overlapping targets, 5027 proteins with at least one cis-acting association signal were retained for instrumental variable selection. For proteins available in both datasets, UKB-PPP (Olink) instruments were prioritised as the primary plasma pQTL source given the larger sample size.^23^

To extend pQTL coverage to East Asian ancestry, plasma pQTL summary statistics from the CKB were incorporated. In CKB, 2923 plasma proteins were quantified using an Olink proteomic panel in 3974 Chinese adults, and genome-wide analyses identified 1312 cis-pQTLs for 1264 proteins.^24^

For eQTL data in European ancestry, whole-blood eQTL summary statistics from the eQTLGen Consortium were used, comprising 31,684 individuals from 37 predominantly European cohorts.^25^ In eQTLGen, gene expression in whole blood or peripheral blood cells was quantified using RNA microarrays or RNA sequencing, and genotypes were obtained using genome-wide SNP arrays or sequencing platforms. Associations between genetic variants and transcript abundance were then tested to identify significant eQTLs.

For East Asian transcript instruments, whole-blood cis-eQTL summary statistics were obtained from the Japan COVID-19 Task Force (JCTF), which generated genotype data together with whole-blood mRNA profiles in 1405 Japanese participants. Fine-mapping of cis variant-gene associations was conducted in 1019 RNA-sequenced samples, yielding a catalogue of 3464 high-confidence putative causal cis-eQTLs.^26^

### Genetic instrument selection

Genetic instruments were derived from cis- QTLs for proteins and gene expression. For plasma proteins, lead cis-pQTLs were selected from UKB-PPP and deCODE, and additionally from CKB for East Asian ancestry. Cis-pQTLs were restricted to variants within ±1 Mb of the corresponding protein-coding gene and associated with protein concentrations at genome-wide significance (*P* < 5 × 10^−8^). For transcript instruments, lead cis-eQTLs were obtained from the eQTLGen whole-blood meta-analysis for European ancestry and from the JCTF whole-blood cis-eQTL resource for East Asian ancestry. Cis-eQTLs were defined as variants within 1 Mb of the corresponding gene reaching genome-wide significance (*P* < 5 × 10^−8^). Across all QTL resources, linkage disequilibrium (LD)-based clumping was applied (r^2^ < 0.001) to retain independent instruments, and instrument strength was assessed using F-statistics, with weak instruments excluded (F ≤ 10). Variants unavailable in the outcome GWAS, variants with incompatible alleles, and palindromic variants with ambiguous strand orientation were excluded during harmonisation. The resulting instrument sets were carried forward to Mendelian randomisation analyses.

For bidirectional Mendelian randomisation (MR) analyses between cataract and cataract-related traits (non-QTL phenotypes), instruments were selected as genome-wide significant variants from ancestry-matched GWAS summary statistics (*P* < 5 × 10^−8^), with LD clumping, harmonisation, and F-statistic filtering applied using the same thresholds as for the QTL instruments.

### Statistical analysis

#### Genome-wide association analysis

Genome-wide association testing was performed using REGENIE (v4.1.2) within the All of Us Researcher Workbench.^27^ REGENIE implements a two-step whole-genome regression that accounts for population structure and cryptic relatedness. In Step 1, a leave-one-chromosome-out polygenic prediction was fit using LD-pruned variants (pairwise r^2^ < 0.1 in 100 kb windows with a 50 kb step). In Step 2, SNPs in the All of Us v8 srWGS ACAF threshold callset were tested for association with senile cataract under an additive genetic model, with approximate Firth penalised likelihood ratio testing applied to variants with logistic regression *P* < 0.05 to account for case-control imbalance (--firth --approx --pThresh 0.05). All models were adjusted for age at enrolment, sex assigned at birth, and the first ten principal components computed within the European-ancestry subset. Genome-wide significance was set at *P* < 5 × 10^−8^.

#### Meta genome-wide association study for cataract

Fixed-effects inverse-variance-weighted meta-analyses were performed separately for European- and East Asian-ancestry cataract GWAS summary statistics (Fig. 1). Meta-analysis was implemented in METAL (release 2020-05-05) using the standard error-based scheme, which aggregates study-specific effect estimates weighted by the inverse of their variance. For each variant, effect alleles were harmonised across cohorts, and per-study association results (beta, standard error, *P* value, sample size, and effect-allele frequency when available) were used as input. Beta value represented the per-allele log odds ratio for cataract. Between-study heterogeneity was evaluated using Cochran’s Q statistic and the corresponding heterogeneity *P* value.

**Fig. 1:**
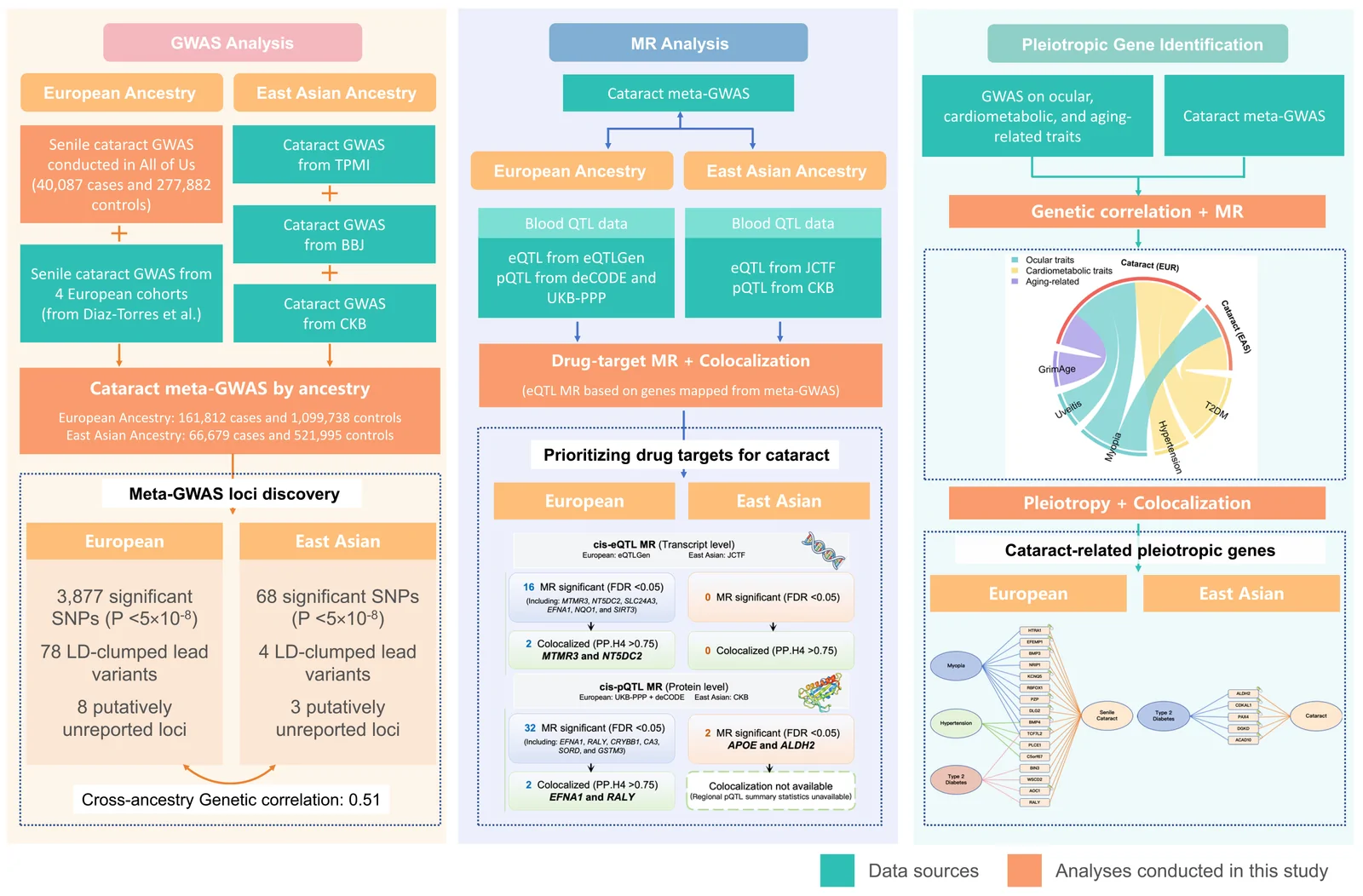
**Study design and workflow.** GWAS, genome-wide association study; eQTL, expression quantitative trait locus; pQTL, protein quantitative trait locus; PP.H4, posterior probability for colocalisation under hypothesis 4; LD, linkage disequilibrium; CKB, China Kadoorie Biobank; TPMI, Taiwan Precision Medicine Initiative; BBJ, BioBank Japan; JCTF, Japan COVID-19 Task Force; UKB-PPP, UK Biobank Pharma Proteomics Project.

To evaluate the quality of the AoU GWAS and meta-GWAS, we calculated standard summary statistic metrics, including the mean chi-square statistic, genomic inflation factor (lambda GC), LD score regression (LDSC) intercept, attenuation ratio, and SNP-based heritability. LDSC analyses were performed using ancestry-specific LD scores. For the binary cataract outcome, SNP-based heritability was reported on the liability scale.

To reduce potential bias arising from cohort-specific variant availability, meta-analyses in both ancestries were restricted to SNPs present in at least two contributing GWAS datasets. In addition, because TPMI reported summary statistics on the GRCh38 build whereas the remaining GWAS datasets were based on GRCh37, genomic coordinates were harmonised by lifting CKB and BBJ variants from GRCh37 to GRCh38 before downstream analyses.

Putatively novel loci in the AoU GWAS and ancestry-specific cataract meta-GWAS were defined as genome-wide significant lead variants located more than 500 kb from previously reported cataract loci in relevant large-scale GWAS, including Choquet et al., Diaz-Torres et al., the MVP senile cataract GWAS, and the East Asian GWAS datasets from TPMI, BBJ, and CKB.^6^

#### Two-sample Mendelian randomisation

Two-sample MR was conducted to estimate the effects of genetically proxied transcript levels and circulating protein levels on cataract risk. Prior to MR, exposure and outcome summary statistics were harmonised to ensure consistent effect-allele alignment and strand orientation. MR analyses were implemented in TwoSampleMR (v0.6.9). The inverse-variance weighted (IVW) approach was used as the primary estimator, with complementary sensitivity analyses including MR-Egger, weighted median, simple mode, and weighted mode where the number of instruments allowed.^28^ When only a single valid instrument was available, MR effects were estimated using the Wald ratio.^29^ MR estimates are presented as odds ratios (ORs), representing the change in cataract odds per one standard deviation unit increase in genetically proxied exposure.

Heterogeneity, horizontal pleiotropy, and directionality were evaluated using standard diagnostic procedures, including Cochran’s Q statistic, and the MR-Egger intercept. Multiple testing was addressed by applying a false discovery rate (FDR) adjustment where applicable.

#### cis-eQTL based MR for locus-mapped genes

To provide mechanistic support for genes mapped to genome-wide significant loci identified in the cataract GWAS meta-analysis, a locus-restricted cis-eQTL based MR analysis was performed. The analysis was restricted to GWAS-mapped genes with eligible cis-eQTL instruments in eQTLGen (European ancestry) or JCTF (East Asian ancestry), with statistical significance determined using FDR.

#### Proteome-wide cis-pQTL MR analysis

To prioritise circulating proteins with genetically supported relevance to cataract, a proteome-wide cis-pQTL MR screen was performed. In European ancestry, proteins with eligible cis-pQTL instruments from UKB-PPP or deCODE were all tested against the European cataract meta-GWAS. In East Asian ancestry, cis-pQTL instruments from CKB were evaluated against the East Asian cataract meta-GWAS. For each ancestry-specific scan, multiple testing was addressed using FDR.

#### Bayesian colocalisation analysis

Bayesian colocalisation was conducted between cataract GWAS loci and QTL signals for genes/proteins prioritised in the MR analyses to strengthen genetic inference and to distinguish colocalised signals from MR associations that could be driven by distinct but correlated variants in LD.^30^ For each target (gene or protein-coding locus), a cis region was defined around the lead QTL variant, and variants within ±500 kb were extracted. Cataract GWAS and QTL summary statistics were harmonised within each region, and analyses were restricted to SNPs jointly available across datasets with matched alleles and effect estimates (and allele frequencies when available). Colocalisation was performed only for targets showing statistically significant MR evidence in the corresponding QTL resource.

Colocalisation was implemented using the approximate Bayes factor approach in the coloc R package, estimating posterior probabilities for five competing hypotheses: no association with either trait (H0), association with the QTL trait only (H1), association with cataract only (H2), association with both traits due to distinct causal variants (H3), and association with both traits driven by a shared causal variant (H4). Strong evidence of colocalisation was defined as PP.H4 >0.75. To avoid unstable estimates, regions with limited variant overlap between the cataract and QTL datasets (fewer than 50 shared SNPs) were excluded.

#### Genetic correlation analysis

We employed the LDSC method to estimate the genetic correlation between related traits and cataract. This approach leverages GWAS summary statistics to quantify the shared genetic architecture between two phenotypes by analysing the relationship between LD scores and SNP test statistics. LDSC quantifies shared genetic architecture by regressing SNP χ^2^ association statistics on LD scores, enabling separation of polygenic signal from inflation due to confounding. Potential sample overlap between GWAS datasets was accommodated via the intercept term, and an unconstrained-intercept model was applied. Analyses were implemented using the ldsc function in the GenomicSEM R package. European- and East Asian- specific LD scores from the 1000 Genomes Project were used as reference panels for the corresponding analyses.

In addition, to complement genetic correlation and evaluate directional genetic associations, bidirectional two-sample MR was performed between cataract and each genetically correlated trait in European and East Asian populations. Instrument selection, harmonisation, primary estimators, and sensitivity analyses followed the same protocol as described above. Effect estimates are presented as odds ratios for categorical outcomes and betas for continuous outcomes (epigenetic clocks).

#### Identification of pleiotropic loci and colocalisation analysis

For trait-cataract pairs showing significant genetic correlation, pleiotropic variants jointly influencing both phenotypes were further prioritised to nominate genetically supported candidates. Genome-wide summary statistics for each correlated trait pair were harmonised at the SNP level. Pleiotropic signals were then identified using the Pleiotropic Analysis under Composite Null Plus (PLACO+) framework, which tests for non-null effects on both traits while accounting for cross-trait correlation or potential sample overlap. Variants achieving genome-wide significance in PLACO+ (*P*_PLACO_ <5 × 10^−8^) and showing additional nominal evidence of association with both cataract and the corresponding trait (*P*_GWAS_ <1 × 10^−4^ for each phenotype) were taken forward and grouped into loci using a distance-based approach (500 kb) to define independent pleiotropic regions.^31^^,^^32^

Within each pleiotropic locus, Bayesian colocalisation was performed to evaluate whether the two traits were consistent with a shared causal variant, using the same colocalisation framework described above. Genes mapped to pleiotropic loci and supported by colocalisation were subsequently assessed for therapeutic relevance by querying the Drug-Gene Interaction Database (DGIdb, v5.0) (https://dgidb.org). Additional implementation details, including estimation of cross-trait Z-statistic correlation and locus definition procedures, are provided in the Supplementary Methods.

### Ethics

The All of Us Research Program protocol was reviewed and approved by the Institutional Review Board of the All of Us Research Program (#2016-05-TN-Master). All participants provided written informed consent at enrolment. The present study used de-identified participant-level data accessed by authorised researchers through the All of Us Researcher Workbench in accordance with the All of Us Data User Code of Conduct and data access policies. Other GWAS and QTL datasets used in this study were publicly available summary-level data from previously published studies or biobank resources. No additional ethics approval or informed consent was required for their secondary use. The study was conducted in accordance with relevant ethical regulations and the ethical principles of the 2024 Declaration of Helsinki.

### Role of funders

The funder had no role in this work.

## Results

### GWAS analysis in the All of Us Research Program

Our GWAS of senile cataract in AoU included 40,087 senile cataract cases and 277,882 controls without any recorded cataract diagnosis, and tested 11,590,523 SNPs. LDSC-based quality-control metrics showed limited test-statistic inflation and low detectable polygenic signal in this single-cohort analysis (Supplementary Text). At the 5 × 10^−8^ threshold, only one SNP reached significance: rs73188175 at *KALRN* (*P* = 4.35 × 10^−8^) (Supplementary Figs. S1 and S2 and Table S4).

Compared with previously reported large-scale cataract GWAS, including Diaz-Torres et al., Choquet et al., and MVP, we defined loci as putatively novel if no genome-wide significant variant had been reported within ±500 kb in these prior studies. Based on this definition, the *KALRN* locus had not been previously reported, suggesting that the AoU GWAS contributed one putatively novel cataract-associated locus.

### Genome-wide meta-analysis for cataract

Large-scale GWAS meta-analyses were conducted separately in European and East Asian ancestry. In European ancestry, a fixed-effects meta-analysis of senile cataract across 5 cohorts included 161,812 cases and 1,099,738 controls, with meta-association statistics available for 7,538,447 variants. In total, 3877 SNPs reached genome-wide significance (*P* < 5 × 10^−8^). The strongest association was observed at rs4814857, annotated to *SLC24A3* (*P* = 2.30 × 10^−86^) (Fig. 2, Supplementary Fig. S2). After LD-based clumping (r^2^ < 0.001), 78 independent genome-wide significant loci were identified (Supplementary Table S5). Of these, 8 lead variants were located more than 500 kb from genome-wide significant loci reported in previous large-scale cataract GWAS, as described above, and were therefore considered putatively novel. All 8 putatively novel lead variants showed directionally concordant effects and no significant between-study heterogeneity. These included loci annotated to *ARHGAP21*, *TSC22D2*, and *OR12D2*, with rs1419636 near *OR12D2* also showing a directionally consistent association in the MVP GWAS dataset (*P* = 0.0008) (Supplementary Table S6). Standard LDSC-based quality-control metrics, including mean χ^2^, λ_GC_, LDSC intercept, attenuation ratio, and SNP-based heritability, indicated no evidence of substantial confounding in the European meta-analysis (Supplementary Text).

**Fig. 2:**
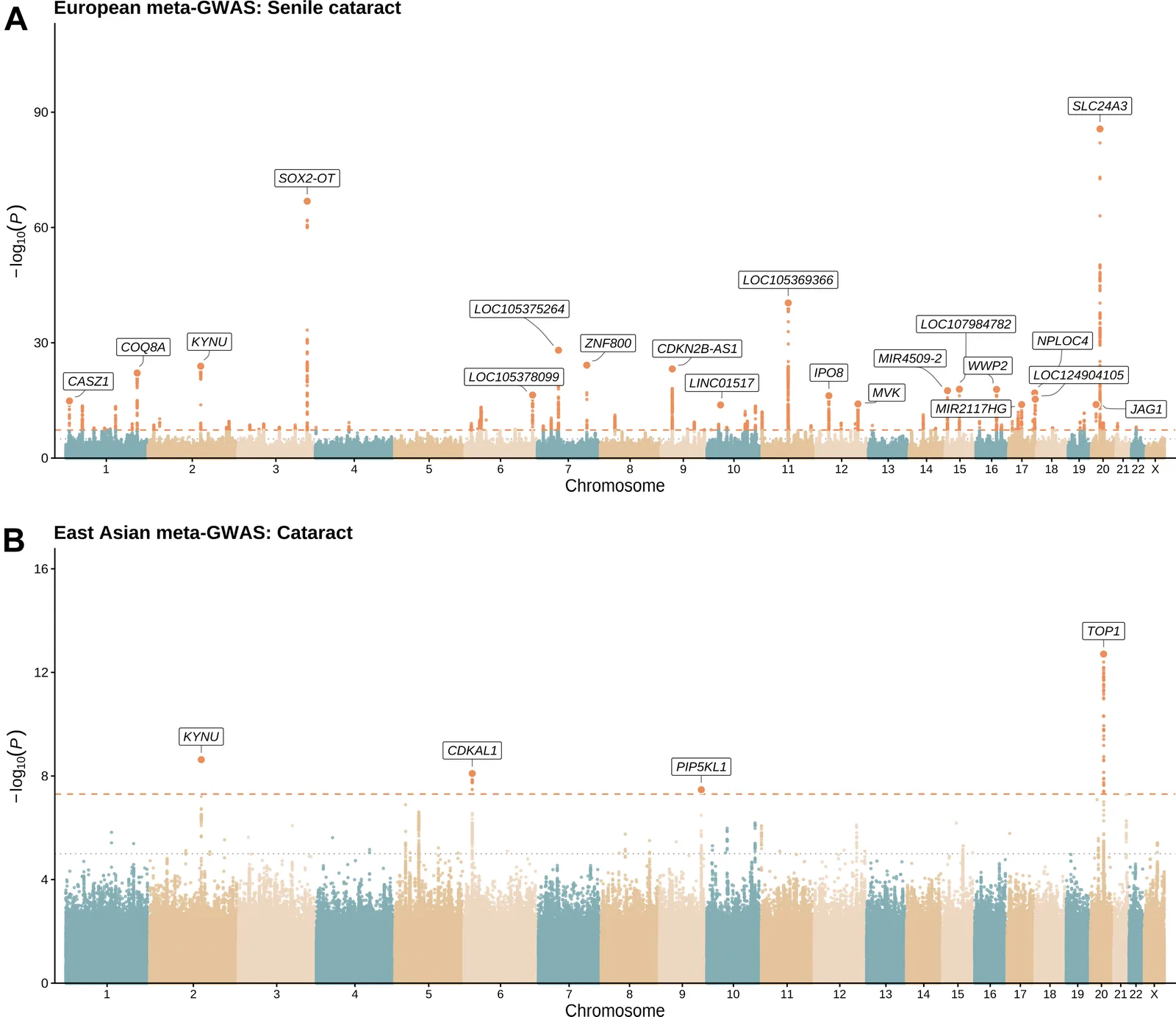
**Genome-wide meta-analysis of cataract in European and East Asian ancestry.** (A) European-ancestry fixed-effects meta-analysis of senile cataract across five cohorts (161,812 cases and 1,099,738 controls). (B) East Asian-ancestry meta-analysis across three cohorts (66,679 cases and 521,995 controls). The horizontal dashed line denotes the genome-wide significance threshold (*P* = 5 × 10^−8^).

In East Asian ancestry, a meta-analysis across three cohorts included 66,679 cases and 521,995 controls, with summary statistics available for 9,713,634 variants. In this analysis, 68 SNPs reached genome-wide significance (*P* < 5 × 10^−8^). The strongest association was observed at rs79779579, annotated to *TOP1* (*P* = 1.96 × 10^−13^) (Fig. 2, Supplementary Fig. S2). After clumping, 4 independent genome-wide significant loci were identified, with no marked between-study heterogeneity observed for the lead variants (Supplementary Table S7). At the locus level, 3 of the 4 loci could be considered novel in East Asians (>500 kb from previously reported significant variants in the GWAS contributing to the East Asian meta-analysis), including loci annotated to *KYNU* (rs7575300), *CDKAL1* (rs9356744), and *PIP5KL1* (rs10987774) (Supplementary Table S8). Quality-control metrics likewise showed no evidence of material test-statistic inflation in the East Asian meta-analysis (Supplementary Text).

The cross-ancestry genetic correlation between European and East Asian meta-analyses, estimated using POPCORN, was 0.51 (*P* = 5.8 × 10^−7^), indicating moderate sharing of cataract genetic architecture between the two ancestry groups.

### Cis-eQTL based Mendelian randomisation and colocalisation for GWAS-mapped genes

To facilitate biological interpretation and prioritise genetically supported GWAS-implicated candidates for cataract, two-sample MR analyses were performed using cis-eQTL instruments. For the European-ancestry cataract meta-analysis, the 3877 genome-wide significant SNPs mapped to 190 genes, of which 106 had eligible cis-eQTL instruments available in the eQTLGen resource. MR was conducted for these genes, and after FDR correction, genetically predicted expression levels of 16 genes were significantly associated with cataract risk, including *MTMR3* and *NT5DC2*, as well as GWAS-proximal genes such as *SLC24A3*, *EFNA1*, and *TSC22D2* (Supplementary Table S9). Bayesian colocalisation was then conducted between cataract GWAS signals and the corresponding eQTL signals. Strong evidence of colocalisation was observed for *MTMR3* and *NT5DC2* (PP.H4 = 0.85 and 0.96, respectively) (Fig. 3, Supplementary Table S10).

**Fig. 3:**
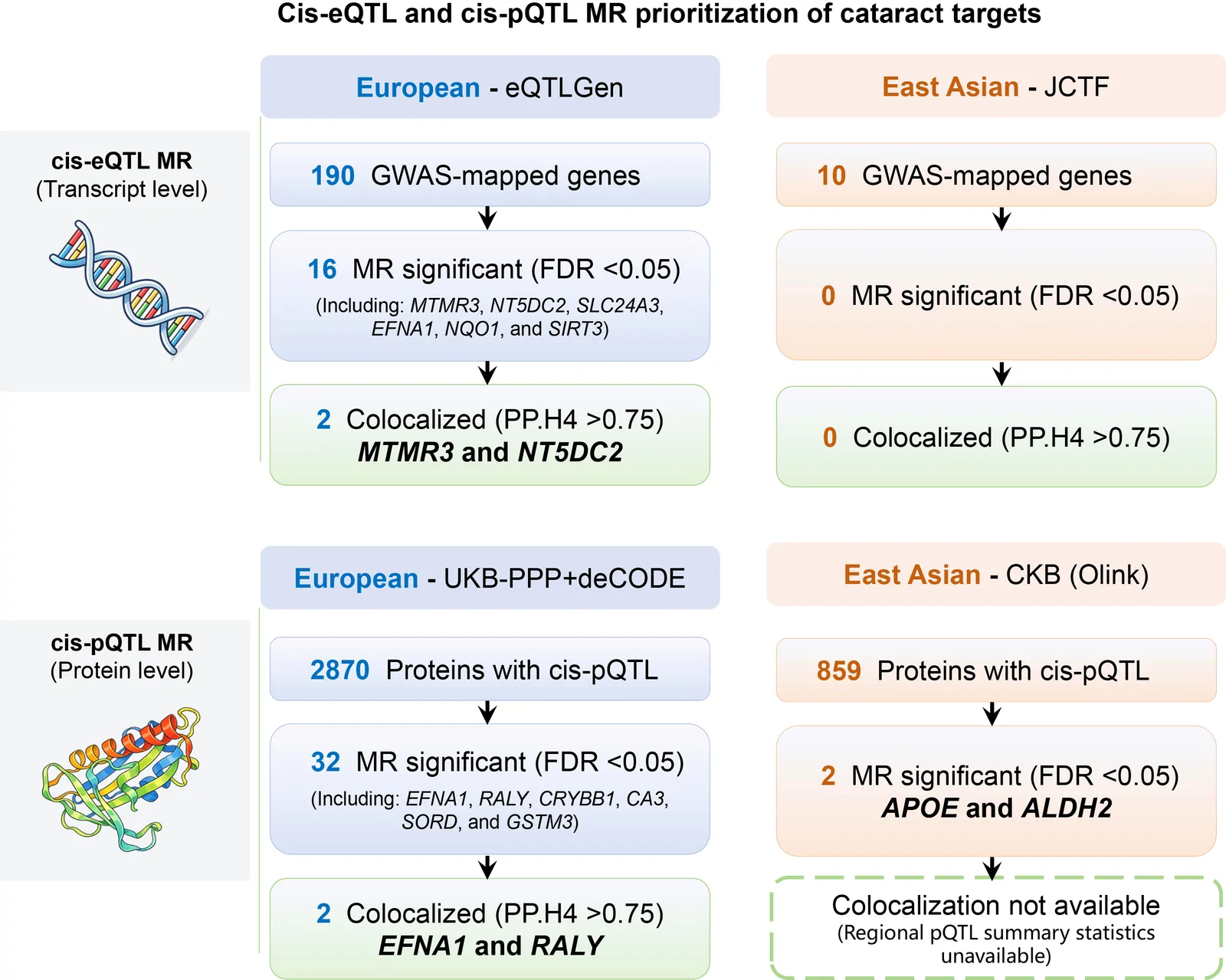
**Overview of cis-eQTL- and cis-pQTL- based MR and colocalisation analyses for cataract in European and East Asian ancestry.** ∗Colocalisation for the CKB pQTL analysis was not performed because regional pQTL summary statistics were unavailable. eQTLGen, eQTLGen Consortium whole-blood meta-analysis; JCTF, Japan COVID-19 Task Force; UKB-PPP, UK Biobank Pharma Proteomics Project; CKB, China Kadoorie Biobank; pQTL, protein quantitative trait loci; eQTL, expression quantitative trait loci.

For the East Asian meta-analysis, the 68 genome-wide significant SNPs mapped to 10 genes, which were taken forward for cis-eQTL based MR using the JCTF whole-blood eQTL resource. However, no significant associations were observed between genetically predicted expression of these genes and cataract risk (Supplementary Table S11).

### Proteome-wide cis-pQTL MR and colocalisation analysis

Proteome-wide two-sample MR analyses were performed separately in European and East Asian ancestry using cis-pQTL instruments to evaluate the effects of genetically proxied circulating protein abundance on cataract risk. In European ancestry, among 2870 proteins with at least one eligible cis-pQTL instrument, 32 proteins were significantly associated with age-related cataract after FDR correction (Supplementary Table S12). The strongest associations were observed for *IGHMBP2*, *ARL4D*, *RALY*, *ITIH5*, *ATF6B*, and *EFNA1*. Notably, 5 proteins were significant in both pQTL resources, providing internal replication of these signals (Supplementary Table S12).

Bayesian colocalisation was subsequently conducted for proteins prioritised by MR to distinguish shared causal signals from associations driven by linkage disequilibrium. Strong evidence of colocalisation with cataract GWAS signals was further observed for 2 proteins, *EFNA1* and *RALY* (PP.H4 = 0.99 and 0.92, respectively; Fig. 3 and Supplementary Table S13).

In East Asian ancestry, among the 1264 proteins with cis-pQTLs reported in CKB, 859 had at least one eligible instrument available for the East Asian cataract meta-GWAS. After FDR correction, only 2 proteins, *APOE* and *ALDH2*, were significantly associated with cataract risk (Supplementary Table S14). Colocalisation analysis could not be performed for the CKB pQTL results because full pQTL summary statistics were not available.

### Genetic correlations between cataract and related traits

Using LDSC, genome-wide genetic correlations were estimated between cataract and related traits, including myopia/refractive error, uveitis, T2DM, hypertension, and epigenetic ageing, based on ancestry-matched GWAS summary statistics. After Bonferroni correction, significant correlations were observed with myopia, T2DM, and hypertension in European ancestry and with T2DM in East Asian ancestry. Nominally significant correlations were observed with uveitis and GrimAge in Europeans and a negative correlation with refractive error (proxy for myopia) in East Asians (*P* < 0.05, Fig. 4, Supplementary Table S15). Among the significant cataract-trait pairs, the strongest genetic correlation was observed between cataract and T2DM (r_g_ = 0.49, *P* = 5.18 × 10^−14^).

**Fig. 4:**
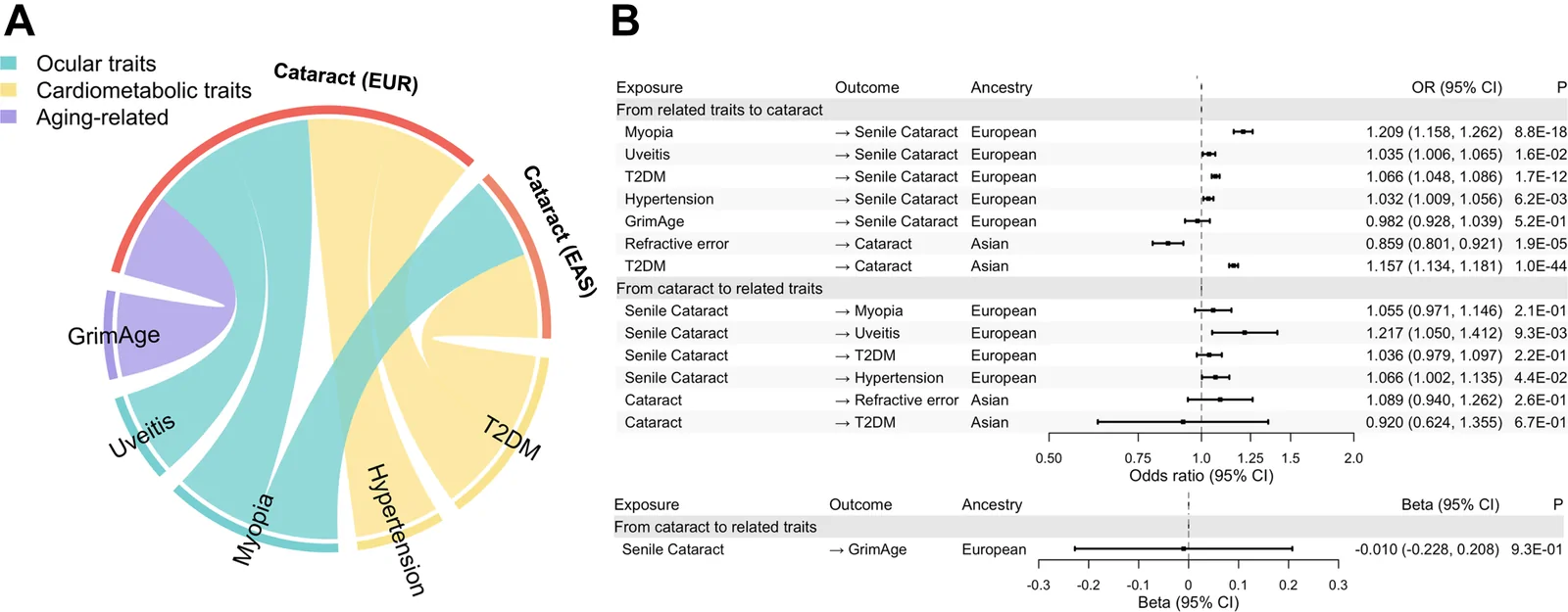
**Genetic correlations and MR results for cataract and related traits.** (A) Genetic correlations between cataract and related traits. (B) Bidirectional MR results for cataract and related traits. ∗Genetic correlations with *P* < 0.05 are shown. ∗MR effect estimates are shown as odds ratios (ORs) for binary outcomes and as beta coefficients when the epigenetic clock measure was analysed as a continuous outcome. ∗“Myopia” denotes clinically defined myopia in European ancestry, whereas “Refractive error” was used as a proxy for myopia in East Asian ancestry. East Asian estimates were retained on the original refractive-error scale, with lower values indicating greater myopic liability. T2DM, type 2 diabetes.

### Bidirectional Mendelian randomisation between cataract and genetically correlated traits

To complement the genetic correlation results and assess directionality, bidirectional two-sample MR was performed for genetically correlated trait-cataract pairs in European and East Asian ancestry. After Bonferroni correction (European: *P* < 0.05/5; East Asian: *P* < 0.05/2), only the cataract-to-uveitis association was significant in the cataract-to-related-trait direction in Europeans. In the reverse direction, genetically proxied myopia, T2DM, and hypertension liability were significantly associated with senile cataract risk. In East Asian ancestry, lower genetically proxied refractive error, indicating greater myopic liability, and genetically proxied T2DM liability were significantly associated with cataract risk, while the reverse analyses were not statistically significant (Fig. 4, Supplementary Table S16).

### Pairwise pleiotropic loci and genes for cataract and genetically correlated traits

Building on the genetic correlation results, PLACO+-based pleiotropy analyses were performed for 5 cataract-trait pairs. PLACO+ was used to identify variants with evidence of horizontal pleiotropy at genome-wide significance (*P*_PLACO_ <5 × 10^−8^), further requiring nominal evidence of association with each phenotype (*P* < 1 × 10^−4^ for both the cataract-related trait and cataract) to focus on variants showing effects on both traits. Among ocular traits, 832 and 431 pleiotropic SNPs were identified for cataract-myopia and cataract-uveitis, respectively, in European ancestry, whereas no significant pleiotropic signals were detected for either pair in East Asian ancestry (Supplementary Tables S17 and S18). For ageing-related traits, the cataract-GrimAge analysis identified 20 pleiotropic lead variants at genome-wide significance (Supplementary Table S19). For cardiometabolic traits, pleiotropic signals were most evident in European ancestry, with 1901 pleiotropic SNPs identified for the cataract-T2DM pair and 251 pleiotropic SNPs for the cataract-hypertension pair (Supplementary Tables S20–S22). In East Asian ancestry, we additionally identified 243 pleiotropic SNPs for the cataract-T2DM pair (Supplementary Tables S23 and S24).

Pleiotropic SNPs were subsequently grouped into genomic intervals for each trait pair and evaluated using Bayesian colocalisation. Strong evidence of colocalised genetic signals was observed for cataract with myopia and two cardiometabolic traits (T2DM and hypertension) in Europeans across 28 intervals, mapping to 24 unique genes (Fig. 5, Supplementary Tables S25–S27). For the cataract-T2DM pair, 4 of the 8 significant pleiotropic genes in European ancestry were supported in the MVP T2DM sensitivity analysis (*TCF7L2*, *AOC1*, *WSCD2*, and *RALY*) (Supplementary Table S28).

**Fig. 5:**
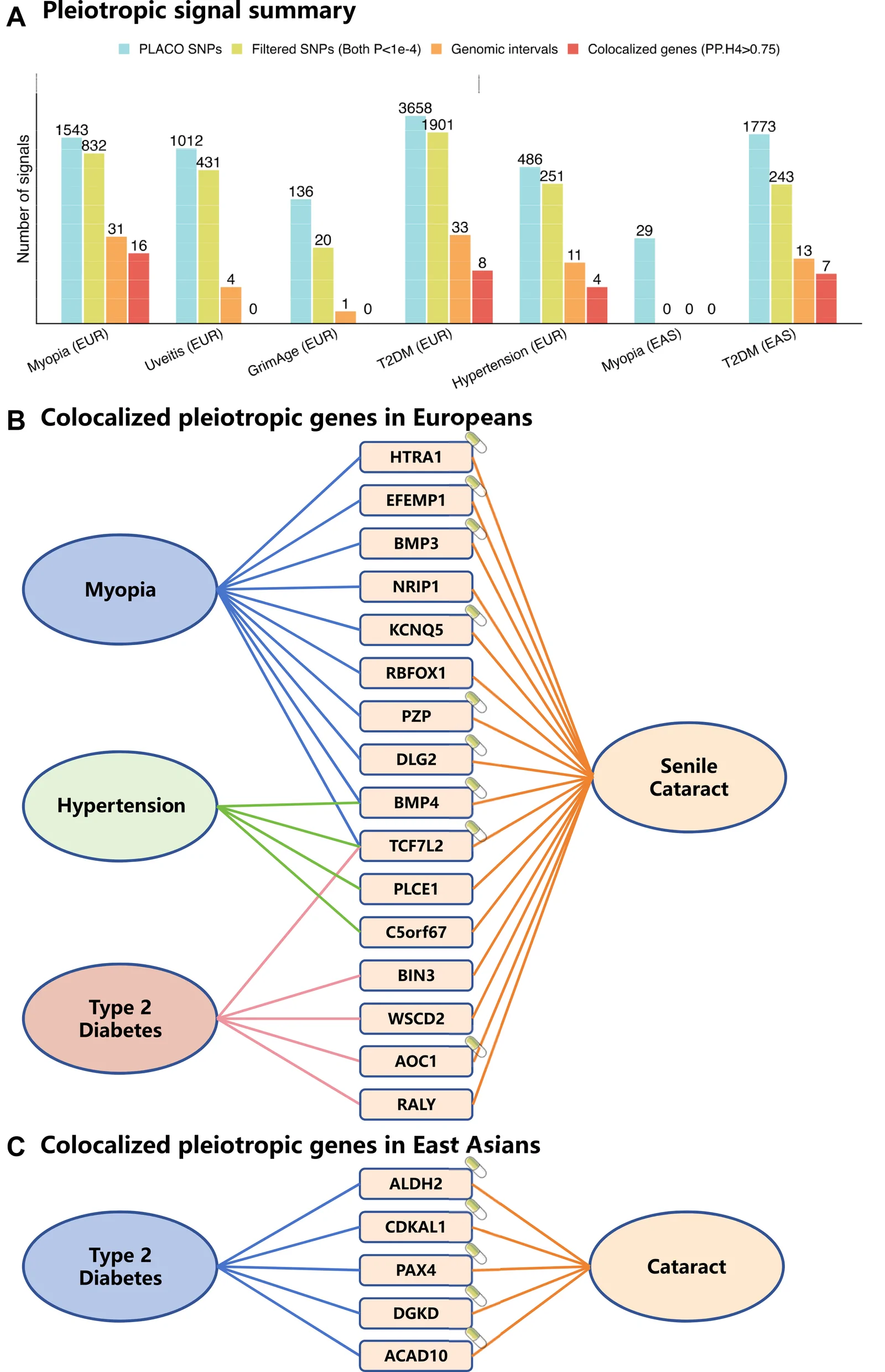
**PLACO identifies pleiotropy and colocalised genes linking cataract with related diseases.** (A) Summary of PLACO-based pleiotropy results across cataract-trait pairs. Bars indicate the number of candidate pleiotropic SNPs identified by PLACO+, the resulting genomic intervals defined from pleiotropic signals, and intervals supported by colocalisation. “Filtered SNPs” denote PLACO-significant variants (*P*_PLACO_ <5 × 10^−8^) that also show nominal evidence in each trait GWAS (*P* < 1 × 10^−4^ for each trait). “Genomic intervals” denote independent pleiotropic intervals obtained by clustering filtered SNPs within 500 kb (colocalisation was performed within the corresponding locus window). (B) Colocalised trait-gene network for cardiometabolic pleiotropy in European ancestry. (C) Colocalised trait-gene network for cardiometabolic pleiotropy in East Asian ancestry. ∗Genes shown in panels B and C had stronger colocalisation support (PP.H4 >0.85) and interpretable gene annotation; non-protein-coding RNA genes were not displayed in the network for readability but were retained in the Supplementary Tables. The pill icon indicates genes with at least one known or potential drug-gene interaction annotated in DGIdb (v5.0).

In East Asian ancestry, colocalisation analysis further supported 7 pleiotropic genes for the cataract-T2DM pair, of which 4 were supported in the KoGES T2DM sensitivity analysis (*CDKAL1*, *ALDH2*, *ACAD10*, and *PAX4*) (Supplementary Tables S29 and S30).

## Discussion

In this study, we first conducted a GWAS of senile cataract in the AoU, including 40,087 cases and 277,882 controls, and identified one putatively novel locus (rs73188175 at *KALRN*). Then we performed large-scale GWAS meta-analyses of cataract in European and East Asian ancestry, respectively. The European-ancestry meta-analysis of senile cataract, combining five cohorts (161,812 cases and 1,099,738 controls), identified 3877 genome-wide significant variants corresponding to 78 independent loci, including 8 putatively unreported lead loci. Several lead variants mapped to genes with prior genetic and functional links to cataract, including *SLC24A3*, *SOX2-OT*, and *BMP4*.^6^^,^^33^, ^34^, ^35^, ^36^ Building on the GWAS signals, we then integrated genes mapped from genome-wide significant loci with cis-eQTL instruments and performed MR, which further highlighted 16 genes with MR evidence supporting effects on senile cataract. Of these, *MTMR3* and *NT5DC2* also showed concordant evidence in colocalisation.

*MTMR3* encodes myotubularin-related protein 3, a phosphoinositide phosphatase involved in autophagy regulation, a process important for organelle degradation during lens fibre cell maturation and maintenance of lens transparency.^37^^,^^38^ *NT5DC2* encodes 5’-nucleotidase domain-containing protein 2 and may promote cell proliferation by stabilising EGFR.^39^ Although direct lens-specific evidence for *NT5DC2* remains limited, EGFR signalling regulates human lens epithelial cell migration, a process relevant to cataract-related lens epithelial remodelling.^40^

To our knowledge, this study represents one of the largest cataract GWAS meta-analyses performed in East Asian ancestry.^41^ By integrating three major East Asian biobanks (66,679 cases and 521,995 controls), we identified 68 genome-wide significant variants. Notably, the genetic correlation between European and East Asian meta-GWAS was 0.51, suggesting that a substantial fraction of cataract susceptibility signals is shared across ancestries despite differences in disease definition, allele frequency, and linkage disequilibrium structure. Among the East Asian loci, the newly identified signal annotated to *PIP5KL1* (rs10987774) was previously identified in a large-scale European cataract GWAS, supporting a cross-ancestry association at this region.^6^ The locus annotated to *KYNU* was also significant in our European-ancestry meta-GWAS. *KYNU* encodes kynureninase, an enzyme in the tryptophan-kynurenine pathway, which is directly relevant to the lens because kynurenine-derived metabolites function as endogenous ultraviolet filters and can contribute to age-related lens protein modification and oxidative damage.^42^, ^43^, ^44^ In contrast, the newly identified locus annotated to *CDKAL1* appears not to have been observed in previous European-ancestry GWAS analyses, suggesting possible ancestry-heterogeneous effects. *CDKAL1* is a well-established T2DM gene, and experimental studies indicate that impaired *CDKAL1* function can reduce glucose-stimulated insulin secretion and compromise β-cell function.^45^ Given the shared genetic background between cataract and T2DM, *CDKAL1* may plausibly influence cataract susceptibility through metabolic pathways related to glycaemic dysregulation.^46^ Together, these cross-ancestry findings underscore the value of expanding cataract genetic studies beyond European-ancestry populations to better capture both shared and ancestry-relevant genetic signals.

Based on the meta-GWAS, we next performed a proteome-wide cis-pQTL MR using pQTL data to identify additional candidate proteins for cataract. Among the 32 proteins prioritised in European ancestry, *EFNA1* and *RALY* were further supported by colocalisation with the cataract association signal. *EFNA1* encodes ephrin-A1, a ligand of EPHA2, and the *EFNA1*-EPHA2 axis has been implicated in lens cell organisation and age-related cataract.^6^^,^^47^ *RALY* encodes an RNA-binding protein, and although direct lens-specific evidence remains limited, RNA-binding proteins are important regulators of lens development and homoeostasis, with several linked to lens defects and cataract.^48^, ^49^, ^50^ From the MR results, *APOC1*, *CA3*, *CRYBB1*, *ITIH1*, and *SORD* were replicated across two independent pQTL resources. Among them, *CRYBB1* and *SORD* provide particularly plausible links to cataract biology. *CRYBB1* encodes βB1-crystallin, one of the major structural crystallins required for maintaining lens transparency, and pathogenic *CRYBB1* variants have been reported to cause inherited cataract.^51^^,^^52^ *SORD* encodes sorbitol dehydrogenase, which converts sorbitol to fructose in the polyol pathway.^53^ Recent studies have highlighted the polyol pathway as a major contributor to diabetic cataract formation through osmotic and oxidative stress in the lens, supporting the relevance of *SORD* to cataract susceptibility.^54^

Using a hypothesis-free design, we also prioritised *APOE* and *ALDH2* as genetically supported proteins in East Asians. *APOE* encodes apolipoprotein E, a central regulator of lipid transport and cholesterol handling, and dysregulated lipid and membrane homoeostasis has been linked to lens ageing and cataract formation.^55^ Apoe-deficient mice also show a high frequency of anterior segment and lens abnormalities, including anterior polar cataracts, consistent with a role for *APOE*-related pathways in maintaining lens transparency.^56^ *ALDH2* encodes mitochondrial aldehyde dehydrogenase 2, a key enzyme that detoxifies reactive aldehydes generated during oxidative stress.^57^ Given the central role of oxidative stress and lipid peroxidation in cataractogenesis, *ALDH2* may plausibly influence cataract susceptibility through aldehyde detoxification and protection of lens epithelial cells from oxidative injury.^58^^,^^59^ However, since full CKB pQTL summary statistics were unavailable, this signal requires further validation in future studies.

Motivated by established risk factors for cataract, we selected representative ocular, cardiometabolic, and ageing-related traits, and estimated their genome-wide genetic correlations with cataract.^9^ In Europeans, we observed evidence of genetic correlations between cataract and all trait groups, consistent with prior findings.^6^^,^^60^ Notably, genetic correlation evidence between cataract and uveitis or epigenetic clock measures has been less commonly reported, and our results extend this literature. In East Asians, T2DM showed a significant genetic correlation with cataract, whereas refractive error (proxy for myopia) showed a nominally significant negative correlation. The lack of significant evidence for the remaining pairs likely reflects more limited statistical power and ancestry differences in allele frequency and LD structure. Subsequent MR analyses provided directional genetic evidence in Europeans for associations of myopia, T2DM, and hypertension with cataract risk, as well as for a cataract-to-uveitis association. Interpretation of the ocular-trait findings should account for phenotype differences. Owing to limited GWAS data, broadly defined myopia and refractive error were used in this study, but neither specifically represents axial myopia. Nuclear cataract-related myopic shift may further complicate interpretation. The cataract-to-uveitis MR association should also be considered exploratory.

We then performed pleiotropy analyses to identify loci shared between cataract and ocular or cardiometabolic traits. Several colocalised genes, including *BIN3*, *RALY*, *CDKAL1*, and *ALDH2*, overlapped with candidate targets highlighted in the GWAS or QTL-based analyses, further supporting their relevance to cataract biology. *BIN3* encodes bridging integrator 3 (Bin3) and loss of Bin3 function in mice causes early-onset cataracts with pronounced lens fibre abnormalities, providing direct in vivo support for its relevance to maintaining lens transparency.^61^ In Europeans, *TCF7L2* emerged across multiple trait pairs, linking cataract with myopia, T2DM, and hypertension, consistent with shared metabolic and Wnt-related genetic mechanisms. *TCF7L2* is a Wnt signalling effector and one of the most established genetic susceptibility loci for T2DM.^62^ *BMP4* connected cataract with myopia and hypertension, and its known role in ocular lens development and lens epithelial cell remodelling provides a plausible biological link.^63^ In East Asians, *CDKAL1* and *PAX4* further pointed to glucose homoeostasis, whereas *AOC1* and *ALDH2* highlighted amine/aldehyde metabolism and related metabolic-oxidative stress pathways, which are implicated in cataractogenesis, particularly in the context of diabetes-related cataract.^64^, ^65^, ^66^, ^67^ However, PLACO + findings for trait pairs with only nominal genetic correlations should also be considered exploratory. These findings warrant further investigation to clarify lens-relevant mechanisms and therapeutic potential.

Several strengths should be noted. First, we conducted a large-scale GWAS meta-analysis of cataract in both European and East Asian populations, providing a more comprehensive view of the genetic architecture of cataract and highlighting cross-ancestry differences that are relevant for genetic prioritisation. Second, by integrating multiple QTL resources and complementary analytic strategies, we prioritised several cataract-associated candidates in both ancestries. Finally, by leveraging shared genetic architecture between cataract and related traits, we identified druggable genes with pleiotropic effects, providing additional candidates for functional validation.

Our study also has several limitations. First, cataract phenotyping was not fully harmonised across contributing cohorts. EHR-coded cataract, ICD-based diagnoses, and cataract surgery represent related but non-equivalent phenotypes and do not allow consistent classification of slit-lamp-graded morphological subtypes. Because only summary statistics were available for the Diaz-Torres et al. GWAS, we could not further harmonise cataract definitions, stratify participants by individual-level sex, race/ethnicity, or genetic ancestry, or conduct subtype-specific analyses. Such heterogeneity may capture partially distinct clinical subtypes and etiologies, and could contribute to differences in discovery yield and locus patterns across ancestries. Nevertheless, given that the Diaz-Torres et al. dataset was reported primarily as European-ancestry, this limitation is unlikely to be the primary driver of the main findings. Second, although we used external GWAS resources and sensitivity analyses where possible, partial sample overlap and phenotype heterogeneity may remain in some related-trait and QTL-based analyses. Third, because QTL resources for ocular tissues remain limited, we were unable to prioritise and validate targets using eye-specific regulatory data. Finally, MR, colocalisation, and pleiotropy analyses provide genetic prioritisation rather than direct functional or therapeutic validation. Therefore, the biological mechanisms and clinical relevance of the prioritised candidates require further experimental and translational investigation.

In conclusion, our large-scale European and East Asian meta-GWAS advances the understanding of cataract genetic architecture. Integrating GWAS with multi-omics QTL resources, and cataract-related trait GWAS prioritised genetically supported candidates including *MTMR3*, *NT5DC2*, *EFNA1*, *RALY*, *APOE*, *ALDH2*, *TCF7L2*, *BMP4*, *CDKAL1*, and *PAX4*. Together, these results provide a cross-ancestry framework for cataract genetic prioritisation and support further validation in lens-relevant tissues and prevention or progression-delay strategies.

## Contributors

X.H.: Conceptualisation, Methodology, Software, Formal Analysis, Visualisation, Writing–Original Draft.

L.Z.: Formal Analysis, Data Verification, Writing–Review & Editing.

V.Z.: Validation, Writing–Review & Editing.

Z.Z., H.T.C., and S.B.: Writing–Review & Editing.

D.G.: Supervision, Writing–Review & Editing.

X.H. and L.Z. accessed and verified the underlying data.

All authors read and approved the final version of the manuscript.

## Data sharing statement

This study used data from the All of Us Research Program’s Controlled Tier Dataset version 8, available to authorised users on the Researcher Workbench. The senile cataract GWAS summary statistics from All of Us and cataract meta-analysis generated in this study will be publicly available on Zenodo upon publication (https://doi.org/10.5281/zenodo.21500725). For senile cataract meta-GWAS data (European-ancestry), summary statistics from Diaz-Torres et al. are available via Zenodo under accession 13594388. MVP senile cataract GWAS summary statistics are available from the NHGRI-EBI GWAS Catalogue (https://www.ebi.ac.uk/gwas/) under accession GCST90477674. East Asian cataract GWAS summary statistics were obtained from publicly accessible biobank resources: TPMI data can be retrieved from the TPMI PheWeb portal (https://pheweb.ibms.sinica.edu.tw/) by searching “cataract”, BBJ data from the BBJ PheWeb portal (https://pheweb.jp/phenotypes) by searching “cataract”, and CKB data from the CKB PheWeb portal (https://pheweb.ckbiobank.org/phenotypes) by searching “H25”.

For molecular QTL resources, whole-blood eQTL summary statistics for European ancestry were obtained from the eQTLGen Consortium (https://eqtlgen.org/). Plasma pQTL summary statistics for European ancestry were obtained from UKB-PPP (Olink), available in Synapse (https://www.synapse.org/Synapse:syn51364943/wiki/622119), and from deCODE proteomics (SomaScan), available from the deCODE download portal (https://download.decode.is/form/folder/proteomics). Whole-blood cis-eQTL summary statistics for East Asian ancestry were obtained from the JCTF (https://humandbs.dbcls.jp/en/hum0343-v3). East Asian plasma pQTL summary statistics from CKB were obtained from the Supplementary files of a preprint (https://www.medrxiv.org/content/10.1101/2023.11.13.23298365v2).

For cataract-related traits, European-ancestry GWAS summary statistics for myopia, uveitis, hypertension, and T2DM were obtained from the GWAS Catalogue under accessions GCST90475880, GCST9047773, GCST90475919, GCST90475667, respectively. Ancestry-specific T2DM GWAS summary statistics from Suzuki et al. are available through the DIAGRAM Consortium website (http://www.diagram-consortium.org/downloads.html). European-ancestry GWAS summary statistics for epigenetic clock traits are available from the GWAS Catalogue under accessions GCST90014288, GCST90014289, and GCST90014292. East Asian GWAS summary statistics for refractive error (proxy for myopia) are available from the YANG Lab portal (https://yanglab.westlake.edu.cn/pubData/). East Asian uveitis GWAS summary statistics can be retrieved from the TPMI PheWeb portal by searching the corresponding phenotype terms. East Asian GWAS summary statistics for T2DM and hypertension are available from the KoGES portal (https://koges.leelabsg.org/pheno) by searching the corresponding phenotype terms.

## Declaration of interests

D.G. is the Chief Executive Officer of Sequoia Genetics, a private company that works with investors, pharmaceutical companies, biotechnology companies, and academic institutions to perform research leveraging genetic data to inform drug discovery and development. L.Z., H.T.C., and S.B. are employees of Sequoia Genetics. The other authors declare no competing interests.
