# Supplementary material for: Cataract GWAS meta-analysis and multi-omics Mendelian randomisation inform disease mechanisms and candidate target prioritisation
Source: eBioMedicine. 2026 Sep 22;132:106485. doi: 10.1016/j.ebiom.2026.106485 (PMC13631533; doi:10.1016/j.ebiom.2026.106485)
Supplement: Supplementary file 1 [file mmc1.docx]

**Supplementary Materials**

**Supplementary Text**

**GWAS summary statistics for cataract-related traits and diseases**

To evaluate shared genetic architecture with cataract and to support candidate target prioritisation, we incorporated GWAS summary statistics for cataract-related traits and comorbid diseases, including myopia/refractive error, uveitis, hypertension, and type 2 diabetes mellitus (T2DM), using ancestry-matched datasets where available.

For European ancestry, GWAS summary statistics for myopia (65,574 cases and 333,242 controls), uveitis (4,512 cases and 443,739 controls), and hypertension (322,448 cases and 105,690 controls) were obtained from MVP. For T2DM, ancestry-specific meta-analysis summary statistics reported by Suzuki et al. were used for the primary analysis, where the European-ancestry meta-analysis aggregated data from 33 European cohorts (242,283 cases and 1,569,734 controls). Because the Suzuki et al. T2DM GWAS may include partial sample overlap with the cataract meta-GWAS, MVP T2DM summary statistics (154,194 cases and 278,454 controls) were used for sensitivity analysis.

For East Asian ancestry, refractive error GWAS summary statistics from Cheng et al. (n =121,172) were used as a proxy for myopia. This meta-analysis included 5 East Asian cohorts and did not include BBJ, CKB, or TPMI. Reported estimates were retained on the original refractive-error scale, with lower values indicating greater myopic liability. Uveitis summary statistics were obtained from TPMI (1,663 cases and 296,346 controls) and were retained only for supportive analysis because no suitable alternative East Asian uveitis GWAS was available. For hypertension, GWAS summary statistics were obtained from KoGES (14,834 cases and 57,395 controls). For T2DM, the Asian-ancestry meta-analysis of 25 cohorts (88,109 cases and 339,395 controls) reported by Suzuki et al. was used as the primary dataset, while KoGES diabetes GWAS summary statistics (5,083 cases and 67,127 controls) were used for sensitivity analysis.

To capture aging-related biology relevant to cataract, GWAS summary statistics for epigenetic clocks were additionally incorporated. Epigenetic clock measures (Hannum, GrimAge, and PhenoAge) were analysed as continuous traits, and summary statistics were available in European ancestry from a GWAS of 34,710 participants.

**Genomic data of All of Us**

Whole-genome sequencing data were generated by the All of Us Genome Centers using short-read whole-genome sequencing (srWGS) with PCR-free library preparation on the Illumina NovaSeq platform to a mean depth of ≥30×, aligned to the GRCh38 reference genome, and jointly called using the Genome Analysis Toolkit (GATK). Comprehensive descriptions of the sequencing pipeline, joint variant calling, and centralised sample- and variant-level quality control procedures have been reported previously^1^. For the current analysis, we used the All of Us v8 srWGS Allele Count/Allele Frequency (ACAF) threshold callset, which includes variants passing centralised quality filters with ancestry-specific allele frequency >1% or allele count >100 in at least one computed ancestry group.

**Sample selection of All of Us**

Analyses were restricted to participants of genetically inferred European ancestry, as assigned by the All of Us centralised ancestry prediction pipeline. Eligible participants were further required to have srWGS data available and complete information on age at enrollment, sex assigned at birth, and the model covariates. After applying these criteria, the final analytic sample comprised 317,969 participants. Among the 48,330 individuals meeting the EHR-based senile cataract case definition, 40,087 (82.9%) met all inclusion criteria and were retained as cases; 277,882 eligible participants with no record of any cataract diagnosis served as controls.

**Principal component analysis**

To adjust for population substructure within the European-ancestry subset, principal components were computed de novo within the EUR sample using PLINK v2.0 on a set of LD-pruned autosomal variants (pairwise r² <0.1 in 100 kb windows with a 50 kb step size). The first ten principal components were retained as covariates in the association model.

**Identification of pleiotropic loci and colocalisation analysis**

Building on the genetic-correlation analyses, pleiotropic variants jointly influencing both phenotypes were identified for selected related trait-cataract pairs using the Pleiotropic Analysis under Composite Null Plus (PLACO+) framework, followed by Bayesian colocalisation to evaluate evidence for shared genetic signals. PLACO+ was used because it allows for cross-trait correlation and potential sample overlap between GWAS datasets.

Briefly, genome-wide GWAS summary statistics for each trait pair were first harmonised at the SNP level to ensure consistent variant representation, effect-allele alignment, and strand orientation across datasets. Variants with unresolved allele mismatches or missing information required for downstream calculations were excluded. For SNPs shared between the two GWAS datasets, Z-statistics were calculated for cataract and the related trait as β/SE. These Z-statistics and corresponding *P* values were then used to estimate the marginal variance of Z-statistics for each trait (VarZ) and the cross-trait correlation of Z-statistics (CorZ). VarZ and CorZ were estimated using the procedures developed by Ray et al., applying a *P*-value threshold of 1×10^-4^ to reduce the influence of strongly associated variants when estimating the background correlation structure.

Using the resulting VarZ and CorZ estimates, PLACO+ was applied genome-wide via the “placo.plus” function to compute a pleiotropy test statistic and P value for each SNP. Variants reaching genome-wide significance in PLACO+ (P <5×10^-8^) were considered pleiotropic candidates. To focus on variants showing evidence of association with both phenotypes, PLACO+-significant variants were further restricted to those with nominal evidence of association with both cataract and the related trait (P <1×10^-4^ for each phenotype).

To define pleiotropic loci, filtered variants were ordered by chromosome and physical position and grouped using a 500 kb distance criterion to delineate approximately independent pleiotropic regions. For each region, the minimum and maximum SNP positions were recorded, and the colocalisation window was defined as this interval expanded by ±250 kb. Bayesian colocalisation was then performed within each locus window using the approximate Bayes factor approach implemented in the coloc R package. Posterior probabilities were estimated for five hypotheses (H0-H4), and strong support for a shared genetic signal was defined as PP.H4 >0.75. To ensure robust inference, loci with fewer than 50 intersecting SNPs between the two datasets within the tested window were excluded from colocalisation.

**Quality assessment of GWAS**

To assess the quality of the GWAS conducted in this study, we calculated standard LDSC-based quality-control metrics.

For the AoU GWAS, the mean χ² statistic was 1.07, with a genomic inflation factor (λ_GC_) of 1.07 and an LDSC intercept of 1.06 (SE =0.01). The SNP-based heritability was 0.017 on the liability scale (SE =0.011). Given the low mean χ² statistic, the attenuation ratio was not considered informative.

For the European meta-GWAS, the mean χ² statistic was 1.38, λ_GC_ was 1.27, and the LDSC intercept was 1.04, with an attenuation ratio of 0.11. These results indicate that the observed inflation was largely attributable to polygenic signal rather than uncontrolled confounding. The SNP-based heritability was estimated at 0.032 (SE =0.002).

For the East Asian meta-GWAS, the mean χ² statistic was 1.02, λ_GC_ was 1.02, and the LDSC intercept was 0.98, indicating no evidence of material test-statistic inflation. The SNP-based heritability was estimated at 0.011 (SE =0.002).

**Supplementary Figure**

**Supplementary Figure 1 Genome-wide association analysis of senile cataract in All of Us**

**Supplementary Figure 2 QQ plots for cataract GWAS**

QQ plots for (A) senile cataract in All of Us, (B) senile cataract in European ancestry meta-GWAS, and (C) cataract in East Asian ancestry meta-GWAS.

**Reference**

1.	All of Us Research Program Genomics Investigators. Genomic data in the All of Us Research Program. *Nature.* 2024;627(8003):340-46.
